# A Pilot Study to Assess Safety and Feasibility of Intrathecal Immunoglobulin for the Treatment of Adults with Tetanus

**DOI:** 10.4269/ajtmh.18-0153

**Published:** 2018-06-18

**Authors:** Huynh Thi Loan, Lam Minh Yen, Evelyne Kestelyn, Nguyen Van Hao, Nguyen Thi Hoang Mai, Duong Bich Thuy, Ha Thi Hai Duong, Nguyen Thi Phuong Dung, Nguyen Hoan Phu, Pham Thi Lieu, Tran Tan Thanh, Ronald Geskus, H. Rogier van Doorn, Le Van Tan, Duncan Wyncoll, Nicholas P. J. Day, Tran Tinh Hien, Guy E. Thwaites, Nguyen Van Vinh Chau, C. Louise Thwaites

**Affiliations:** 1 Hospital for Tropical Diseases, Ho Chi Minh City, Vietnam;; 2 Oxford University Clinical Research Unit, Hospital for Tropical Diseases, Ho Chi Minh City, Vietnam;; 3 Centre for Tropical Medicine and Global Health, University of Oxford, Oxford, United Kingdom;; 4 University of Medicine and Pharmacy, Ho Chi Minh City, Vietnam;; 5 Guy’s and St. Thomas’ Hospitals NHS Trust, London, United Kingdom;; 6 Mahidol Oxford Research Unit, Bangkok, Thailand

## Abstract

Tetanus remains a significant burden in many low- and middle-income countries. The tetanus toxin acts within the central nervous system and intrathecal antitoxin administration may be beneficial, but there are safety concerns, especially in resource-limited settings. We performed a pilot study to assess the safety and feasibility of intrathecal human tetanus immunoglobulin in five adults with tetanus before the conduct of a large randomized controlled trial. Intrathecal injection via lumbar puncture was given to all patients within a median 140 (range 100–165) minutes of intensive care unit (ICU) admission. There were no serious adverse effects associated with the procedure although three patients had probably related minor adverse events which resolved spontaneously. Median ICU length of stay was 14 (range 5–17) days. Two patients required mechanical ventilation and one developed a deep vein thrombosis. Within 240 days of hospital discharge, no patients died and all patients returned to work.

Tetanus is a vaccine-preventable disease which remains a problem in many low- and middle-income countries (LMICs). Mortality risk from tetanus in these settings remain high: a recent review from Africa reported overall mortality risk of 43%.^1^ In many other LMICs with better access to intensive care services, mortality is lower, but the disease continues to cause significant morbidity and resource use.^2^

Symptoms of tetanus are due to the effects of a potent neurotoxin which acts within the central nervous system. Recommended treatment includes intramuscular antitoxin to neutralize unbound tetanus toxin. Early studies in animals reported beneficial effects of giving antitoxin directly into the central nervous system; a practice then tried in humans with reported benefits.^3,4^ A number of clinical trials of intrathecal antitoxin administration in humans have subsequently been performed, which have suggested it may speed disease resolution and prevent complications.^5,6^ However, few trials have been conducted to current standards and only one blinded study has been performed.^7^ In addition, there are few details on the antitoxin preparations used and no detailed descriptions of how intrathecal injections were performed. Importantly, few studies have followed up patients systematically or beyond hospital discharge. Most authors report few adverse events although two cases of reversible paraplegia after high dose (1,500–2,000 IU) of antitoxin with mercury/alcohol preservative were reported.^8^ Recent studies have used lower doses of preservative-free preparations without complications. In view of this limited information, more data are needed before clinical recommendations about the use of intrathecal antitoxin in tetanus can be made.^9^ We, therefore, conducted this open-label pilot study to assess the safety and feasibility of a specific intrathecal antitoxin administration regimen before the planned conduct of a large phase-3 blinded randomized controlled trial. Specifically, we aimed to see if our proposed regimen could be administered and to detect any adverse events.

Adults ≥ 16 years old with tetanus admitted to the intensive care unit (ICU) at the Hospital for Tropical Diseases, Ho Chi Minh City, were eligible for study entry. Exclusion criteria were designed to be consistent with a subsequent randomized controlled trial with requirement for ventilation as primary endpoint and, therefore, included: already received antitoxin, having a contra-indication to antitoxin or lumbar puncture; already receiving mechanical ventilation or expected to before intrathecal injection; pregnancy; and failure to give informed consent. This was the first use of Tetagam-P in Vietnam, and to maximize safety, patients were enrolled sequentially in the study, that is, patients were only enrolled after the preceding patient had been discharged from ICU and the study data monitoring and safety board had authorized continuation. Patients were enrolled pragmatically to maximize safety; thus, once a patient had been discharged and continuation approved, the next eligible patient admitted during working hours (8–4 pm Monday–Friday) was enrolled.

All patients received standard treatment of tetanus with intravenous metronidazole and wound debridement. Spasm control was achieved using intravenous diazepam or midazolam. If spasms were not controlled, then tracheostomy was performed and a non-depolarizing neuromuscular blocking agent, pipecuronium, was given and titrated against spasms. Additional indications for tracheostomy were excessive sputum or laryngeal spasm.

Human tetanus immunoglobulin (Tetagam-P; CSL Behring, Hattersheim am Main, Germany) was given to all patients: 3,000 IU intramuscularly followed by 500 IU intrathecally via lumbar puncture. Both were to be given within 6 hours of ICU admission. This dose was chosen based on previously published studies.^5^ Lumbar puncture was performed in the lateral decubitus position and, if necessary, bolus doses of benzodiazepines and fentanyl were given 5–10 minutes beforehand to allow positioning and control spasms. Intrathecal injections were given using a 20-gauge spinal needle via a 0.2 micrometer filter (Braun Medical, Inc., Melsungen, Germany). Two milliliters of cerebrospinal fluid were removed before injecting the antitoxin as this equates to the volume of Tetagam-P injected intrathecally and has previously been reported to reduce headache incidence.^10^ Patients remained in the supine position for 4 hours following the procedure and were monitored regularly, including careful neurological examination at 1 hour.

Patients were followed daily for all adverse events. For completeness and to reduce bias, we recorded all adverse events regardless of grade or severity. These were defined according to the Common Terminology Criteria for Adverse Events as “any untoward medical event that occurs to a study participant during the course of the study” and followed their grading (grade 1: mild to grade 4: severe or life-threatening).^11^ Thus, events such as nasogastric tube insertion or tracheostomy were included although they may be considered part of management or disease progression. Other end-points included requirement for mechanical ventilation, duration of ICU/hospital stay, in-hospital and 240-day mortality and disability, total dose of benzodiazepines, and neuromuscular blocking agents.

Five patients were enrolled between February and May 2017. Baseline data are given in Table 1. Intramuscular injections were given at a median 90 (range 80–105) minutes after ICU admission. Intrathecal injections were possible in all patients and given a median 140 (100–165) minutes after ICU admission. Two patients reported headache following intrathecal injection, one of whom vomited. These symptoms resolved without intervention, and there were no focal neurological signs observed in either patient. One patient had an episode of chills approximately 2.5 hours after intrathecal injection (3 hours after intramuscular injection), which also resolved without intervention.

**Table 1:** Baseline characteristics of patients (*N* = 5)

	*n* (%) or median (range)
Male	5 (100%)
Age	55 (40–57)
Ablett score on admission^19^*	2 (1–2)
APACHE II score^20^†	4 (3–6)
SOFA score^21^‡	0 (0–0)
Tetanus severity score^22^	4 (−5−11)
Time from first symptom to hospitalization (days)	4 (2–6)
Time from hospital admission to ICU admission (minutes)	21 (18–29)
Time from ICU admission to IM antitoxin (minutes)	90 (80–105)
Time from ICU admission to intrathecal antitoxin (minutes)	140 (100–165)

* Ablett Score: Grade 1, no spasms; Grade 2, spasms not interfering with respiration; Grade 3, severe spasms interfering with respiration; and Grade 4, as Grade 3 but with autonomic nervous system dysfunction.^19^

† Acute Physiology and Chronic Health Evaluation Score.^20^

‡ Sequential Organ Failure Score.^21^

Data regarding clinical course are given in Table 2. Two patients required paralysis and mechanical ventilation for spasm control. Twelve further adverse events were recorded, two of which were thought to be possibly related to the study intervention (Table 2). One patient with mild tetanus, who had not received thrombo-prophylaxis, suffered a deep vein thrombosis (DVT), and a fever of 38°C was recorded in another patient on the same day as antitoxin administration.

**Table 2:** Hospitalization and follow-up data (*N* = 5 unless otherwise stated)

	Median (range) or *n* (%)
Tracheostomy	2 (40%)
Mechanical ventilation	2 (40%)
Duration ventilation (days)*	14 (13–15)
Duration tracheostomy (days)*	15 (14–16)
Autonomic nervous system dysfunction	0 (0%)
Total diazepam (mg)	525 (240–1,030)
Duration diazepam (days)	16 (14–27)
Total midazolam (mg)	1,709 (0–2,321)
Duration midazolam (days)	11.5 (0–16)
Total pipecuronium (mg)*	508 (321–695)
Duration pipecuronium (days)*	13 (12–14)
Total fentanyl (mcg)	0 (0–150)
Ventilator associated pneumonia	0
ICU length of stay (days)	14 (5–17)
Hospital length of stay (days)	27 (16–30)
Adverse events probably due to study intervention	Headache (2 cases)
	Vomiting (1 case)
	Chills (1 case)
Adverse events possibly due to study intervention	Deep vein thrombosis (1 case)
	Fever 38° (1 case)
Adverse events unlikely to be due to study intervention	Phlebitis (3 cases)
	Nasogastric tube (2 cases)
	Hoarse voice after extubation (1 case)
	Tracheostomy (2 cases)
	Ventilation (2 cases)
Alive at hospital discharge	5 (100%)
Alive at 240 days after hospital discharge	5 (100%)
Back to work normally at 240 days after hospital discharge	3 (60%)
Back to work but reduced hours at 240 days after hospital discharge	2 (40%)
Rankin score at 240 days after hospital discharge	1 (0–2)
Other symptoms at 240 days after hospital discharge	Low exercise tolerance (1 case)
	Sensory abnormality and leg pain DVT (1 case)
	Cough (1 case)

* Figures are given for those receiving mechanical ventilation only (*N* = 2).

At 240-day follow-up, 3/5 patients had returned to work as before, and two were working at reduced hours. The patient who had suffered the DVT still experienced some symptoms of sensory abnormality and pain on walking, and one patient reported fatigue if walking far.

Thus, in our setting, our regimen proved safe and feasible. Importantly, we were able to deliver the intervention easily within the target time in all patients including those with severe spasms. Two cases of headache and one of vomiting occurred after antitoxin administration; all resolved without intervention. Similar adverse events have been reported previously: Vakil reported three cases of vomiting of 60 cases treated, and Miranda-Filho reported five cases of headache during intrathecal injection in 58 patients treated, one of which persisted afterward.^10,12^

Generally, post–dural puncture headache is a common phenomenon. Larger sized needles are associated with increased occurrence, and incidence rates of 40% following spinal anesthesia with 22-gauge needles have been reported.^13^ Incidence may be decreased with smaller-sized non-traumatic needles.^13^ Therefore, it is likely that our use of a relatively large (20-gauge) Whitacre-type needle, the only option available in our setting, may have contributed to the headaches we reported.^13^

We have classified the DVT as possibly related to the intervention as, although this is common in critically ill patients, it is also a recognized side effect of intravenous immunoglobulin. Whether intramuscular/intrathecal tetanus immunoglobulin increases the risk of DVT is unclear, and we are not aware of any specific data concerning DVT and tetanus immunoglobulin.^14,15^ Deep vein thrombosis has been reported previously in tetanus but not specifically with respect to intrathecal or intramuscular antitoxin.^16^

The 240-day outcomes revealed that all participants were back at work, with two working at reduced capacity. Tetanus is a critical illness, and although little is published about long-term outcome, it may be that prognosis is similar to other ICU survivors, with a significant number failing to return to work 1 year after hospital discharge.^17,18^ Furthermore, despite the well-known difficulties of performing long-term follow-up in resource-limited settings, we were able to contact all patients at 240 days. Similar follow-up rates in a larger trial would be important to accurately quantify long-term outcome and complications.

Following the results of this pilot study, showing the feasibility of intrathecal antitoxin administration in our setting, we have started a large blinded randomized controlled trial (planned sample size of 272 adults). This study also combines a factorial design to compare human and equine intramuscular antitoxin with requirement for ventilation as a primary endpoint. This study will also look at the long-term outcome and acceptability of the intervention to patients. It is hoped that the results of this study will provide a strong evidence base for future recommendations concerning intrathecal antitoxin therapy in tetanus.
